# Current status of KAB in health education for Chinese college students and SEM validation exploration

**DOI:** 10.3389/fpubh.2025.1671692

**Published:** 2025-09-26

**Authors:** Yuehui Zhao, Yangyang Guan, Zihao Chen, Hailin Gong

**Affiliations:** College of Physical Education, Yangzhou University, Jiangsu, China

**Keywords:** knowledge-attitude-behavior model, structural equation model, health education, college students, confirmatory research

## Abstract

**Introduction:**

In the field of health education, the KAB theory is an authoritative theoretical model worldwide; however, there is not much empirical evidence supporting its application in university health education. Therefore, the purpose of this paper is to use data from Chinese universities to test the pathways and universality of the KAB model, as well as to provide certain suggestions for future health education in universities through a situational analysis.

**Methods:**

We used a random sampling method to conduct a questionnaire survey of 4,508 college students from 76 universities in Jiangsu Province (752 from sports majors and 3,756 from non-sports majors). We employed a self-developed four-point scale questionnaire (20 questions on health knowledge, 14 on attitudes, and 10 on behaviors), which demonstrated good reliability and validity (Cronbach’s *α*: 0.958–0.969). Exploratory factor analysis was conducted to extract the dimensions of attitude and behavior, and structural equation modeling (SEM) was used to verify the K → A → B theoretical pathway.

**Results:**

Current status: The awareness of sexual and reproductive health was highest (89.7%–98%), while knowledge of disease prevention was the weakest (awareness of antibiotic hazards was only 57.5%); sports majors scored significantly higher in health knowledge than non-sports majors (e.g., 15.7% higher in disease prevention knowledge). In terms of behavior, the rate of developing exercise habits among non-sports majors (87.6%) was significantly lower than that of sports majors (97.9%), and only 69.6% actively consulted health issues, showing that students are hesitant to talk to their teachers. Model validation: The SEM fit indices were good (GFI = 0.972, RMSEA = 0.065), confirming three key pathways: knowledge → attitude (*β* = 0.42), attitude → behavior (*β* = 0.33), and knowledge → behavior (*β* = 0.55). Multi-group analysis showed that the model has universality across genders and majors, but the pathway from knowledge to behavior was stronger for males (*β* difference +0.11), while the pathway from attitude to behavior was more pronounced for females (*β* difference +0.09).

**Discussion:**

The KAB model is applicable to the college student population, where health knowledge not only directly influences behavior (*β* = 0.55) but also has an indirect effect through attitude. We need to focus more on teaching disease prevention, such as the hazards of antibiotics, design behavioral intervention courses for non-sports majors, and we should also notice that students are less willing to seek health advice in person. The study validates the universality of the KAB theoretical pathway, giving solid proof for health education in universities.

## 1 Introduction

The World Health Organization (WHO) pointed out in 1997 that health is not merely the absence of disease but should also include a sound and peaceful state of physical, mental, and social well-being, and that schools play an important role in health education (1). It is evident that school health education has long been an important topic of global concern. With the changing times, China has also placed great emphasis on school health education in recent years, with “Healthy China” becoming a key part of the national development strategy (2). In 2016, the Central Committee of the Communist Party of China and the State Council issued the “Healthy China 2030 Planning Outline,” which clearly stated the need to “increase the intensity of school health education, incorporate health education into the national education system, and make health education an important component of quality education at all stages of education” (3).

In this context, as a crucial time for students’ physical and mental growth, as well as the formation of health literacy and the development of healthy lifestyle habits, it is essential to pay attention to school health education, promote the implementation of health education, and enhance students’ health literacy during the higher education stage (4). Therefore, in 2017, the Ministry of Education issued the “Health Education Guidelines for Colleges and Universities” (hereinafter referred to as the “Guidelines”), which clarified the development pathway of health education in higher education institutions and specified the main content of health education in colleges and universities (5). Additionally, the “Guidelines” also explicitly stated that promoting knowledge transfer and behavior formation should be a fundamental principle for conducting health education in higher education institutions.

It is clear that the transfer of knowledge and the formation of behavior are key steps in developing school health education (6). Evidence has shown that the acquisition of knowledge can lead to changes in attitudes, which in turn leads to the formation of behaviors, thus forming the well-known KAB model in recent years in Japan’s school health education field (7, 8). Although the KAB model is widely cited in the field of health education, its applicability to Chinese college students still lacks empirical support. Existing research mostly focuses on descriptive analyses or simple correlations and has not yet employed structural equation modeling (SEM) to systematically test the path relationships between knowledge, attitudes, and behaviors. Given the unique characteristics of college students, the absence of a university-level KAB model will render health interventions ineffective – empirical studies demonstrate that health education lacking theoretical frameworks increases sedentary behavior rates and psychological distress risk (9, 10). while exacerbating obesity and driving healthcare cost escalation (11, 12). Ultimately compromising students’ long-term health outcomes. Previous studies have also emphasized the importance of KAB. For example, an analysis of sports exercise habits indicates that the transformation of behavior requires the mediating role of demand and satisfaction (9). Similarly, research on dietary decision-making has found that attitude and willingness have a direct impact on behavior. In addition, the Japan Health Learning Promotion Committee has also pointed out in its report investigating elementary, junior high, and high schools nationwide that there is an inevitable connection between health knowledge and learning attitude and behavior, which requires structural analysis (13). Furthermore, in health education during the junior high school stage in China, there is a conclusion that the formation of healthy behavior has a negative impact between attitude and behavior (14). However, these studies have not yet integrated SEM to validate the KAB path in the context of Chinese higher education, leaving a critical gap.

Jiangsu Province is the province with the highest density of higher education institutions in China, with a total of 168 universities and 2.3 million students, accounting for 10.2% of the national college student population (15). Therefore, choosing Jiangsu Province as the research location has certain representativeness. The research results will provide a basis for the theoretical universality of the knowledge-attitude-practice (KAP) model and offer key targets for the design of health education interventions in universities. Given the characteristics of the college student population in Jiangsu Province, this study aims to: describe the current characteristics of health knowledge (K), attitudes (A), and behaviors (B) in this population; verify the proposed KAB theoretical path (knowledge → attitude, attitude → behavior, knowledge → behavior) through SEM, and assess the model fit and path significance. The research results will provide evidence for the theoretical universality of the KAB model and identify key targets for designing health education interventions in colleges.

## 2 Survey subjects and research methods

### 2.1 Subjects

Jiangsu Province is one of the most economically active regions in China, with the highest degree of openness and the strongest innovation capability. It holds a crucial strategic position in the overall modernization and comprehensive openness of the country, making it fairly representative (16, 17). Therefore, the survey focuses on 76 provincial universities in Jiangsu.

### 2.2 Methods

This study employed a random sampling method. Teachers responsible for monitoring the physical fitness of college students at these universities conducted an online questionnaire survey using the Tencent survey platform. We collected a total of 4,508 valid questionnaires. Among them, there were 2,188 male students and 2,320 female students based on gender. Based on their majors, there were 752 students studying physical education and 3,756 students studying other majors (see Table 1). The survey ran from August 2023 to February 2024.

**Table 1:** Characteristics of survey participants.

Subject	Number of people (*n*)	Percentage (%)
Total participants	4,508	100.0
Gender		
Males	2,188	48.5
Females	2,320	51.5
Field of study		
Sports majors	752	16.7
Non-sports majors	3,756	83.3

This study designed a questionnaire based on the relevant content and requirements of the “Guidelines” from the Ministry of Education of China, referencing related literature and through expert discussions, and tested its applicability and accuracy through a pre-experiment (13, 14, 18). The main content of the questionnaire includes basic information and covers three main areas: health knowledge, health attitudes, and health behaviors. Health knowledge includes five main areas: “healthy lifestyle,” “disease prevention,” “mental health,” “sexual and reproductive health,” and “safety, emergency preparedness, and risk avoidance,” which consists of 20 questions. It includes 14 questions on health attitudes and 10 on health behaviors, for a total of 44 questions. All questions use a four-point Likert scale: health knowledge (1 = very well, 2 = somewhat well, 3 = not very well, 4 = not at all), health attitudes (1 = strongly agree, 2 = agree, 3 = somewhat disagree, 4 = strongly disagree), and health behaviors (1 = often do, 2 = occasionally do, 3 = rarely do, 4 = never do).

To analyze the current status of KAB in health education in universities, relevant data was first organized. In the current status of health knowledge, the awareness rate of health knowledge is calculated by merging affirmative options (1 = very knowledgeable, 2 = basically knowledgeable). In the current status of health attitudes, the formation rate is calculated by merging affirmative options (1 = strongly agree, 2 = agree). In the current status of health behaviors, the formation rate is calculated by merging affirmative options (1 = often do, 2 = occasionally do).

Regarding the current status of health knowledge among college students, the “Guidelines” clearly states that the content of health education in universities mainly includes five aspects: “health lifestyle,” “disease prevention,” “mental health,” “sexual and reproductive health,” and “safety emergency and risk avoidance.” Therefore, the analysis is conducted from these five dimensions. The “healthy lifestyles” consists of four items: methods to enhance physical fitness (K_1_), healthy dietary habits (K_2_), the dangers of smoking (K_3_), and knowledge of food safety (K_4_). The “disease prevention” includes four items: knowledge related to flu prevention (K_5_), the dangers of antibiotics to health (K_6_), common methods to assess health status (K_7_), and knowledge of chronic disease prevention (K_8_). The “mental health” consists of four items: manifestations of depression and anxiety (K_9_), the relationship between mental health and physical health (K_10_), promoting positive emotions and alleviating negative emotions (K_11_), and ways to self-regulate anxiety and worries (K_12_). The “sexual and reproductive health” consists of four items: methods to prevent sexual assault (K_13_), knowledge of AIDS prevention (K_14_), effective contraceptive methods (K_15_), and prevention of common sexually transmitted diseases (K_16_). The “safety emergency and risk avoidance” consists of four items: self-rescue and mutual rescue in drowning (K_17_), health and safety risks during travel (K_18_), emergency measures for animal bites and scratches (K_19_), and methods of cardiopulmonary resuscitation and trauma rescue (K_20_).

An analysis of college students’ attitudes towards health education revealed that the current status is consists of three factors: “emotional willingness for learning health knowledge” (hereinafter referred to as “emotional willingness”), “willingness to learn health knowledge” (hereinafter referred to as “learning willingness”), and “recognition of the value of health” (hereinafter referred to as “value recognition”). “Emotional willingness” consists of two items: enjoying learning about health knowledge (A_1_) and finding learning health knowledge interesting (A_2_). “Learning willingness” is made up of six items: it is important to learn health knowledge for a healthy life (A_3_), learning health knowledge is very important (A_4_), learning health knowledge will be useful for future life (A_5_), health knowledge is necessary as part of school learning (A_6_), learning health knowledge can alleviate psychological and physical anxiety and worries (A_7_), and learning health knowledge is enjoyable (A_8_). “Value recognition” consists of six items: learning health knowledge will be useful for entering society in the future (A_9_), maintaining health is more necessary than anything else (A_10_), learning health knowledge can make life healthier (A_11_), maintaining health is important for a happy future (A_12_), health is more important than anything (A_13_), and learning health knowledge contributes to the formation of public health (_A14_).

An analysis of the current status of college students’ health behaviors, based on factor analysis of the data, revealed that health behaviors are composed of three factors: “proactive attention,” “proactive learning,” and “habit formation.” Therefore, the current status of college students’ health behaviors can be analyzed from these three dimensions. The “proactive attention” consists of two items: actively paying attention to health knowledge (B_1_) and regularly checking food expiration dates (B_2_). The “proactive learning” includes four items: valuing flu prevention (B_3_), discussing health issues with classmates (B_4_), self-learning health-related knowledge (B_5_), consulting teachers about health issues (B_6_), and browsing health information (B_7_). The “habit formation” is consists of three items: having a habit of exercising (B_8_), consciously exercising to enhance physical fitness (B_9_), and consciously forming good daily routines (B_10_).

### 2.3 Reliability and validity analysis

The analysis of reliability and validity was conducted using a total sample size of 4,508, with Cronbach’s *α* coefficient and Kaiser–Meyer–Olkin (KMO) measure and Bartlett’s test analysis performed separately. The reliability of the health knowledge questionnaire, as measured by Cronbach’s *α* coefficient, is 0.958; the reliability of the health attitude questionnaire is 0.969; and the reliability of the health behavior questionnaire is 0.920. According to relevant research standards, an *α* coefficient less than 0.05 indicates poor internal consistency, above 0.7 indicates acceptable internal consistency, and above 0.8 indicates very high internal consistency (19). So, we can say that the internal consistency of the health knowledge, health attitude, and health behavior questionnaires is very high. Additionally, we checked the structural validity of the questionnaire using the KMO measure and Bartlett’s test of sphericity. The KMO coefficient for health knowledge is 0.954, for health attitude is 0.968, and for health behavior is 0.928, and all of them were significant at *p* < 0.001. According to relevant research standards, a KMO value closer to 1 means better structural validity, while a coefficient below 0.5 indicates poor structural validity (19). Therefore, we can conclude that the structural validity of the questionnaire in terms of health knowledge, health attitude, and health behavior is very good.

### 2.4 Statistical analysis

First, regarding health knowledge, the topics were categorized into five dimensions based on their content: “Healthy Lifestyle,” “Disease Prevention,” “Mental Health,” “Sexual and Reproductive Health,” and “Safety and Emergency Preparedness.” We conducted factor analysis on health attitudes and behaviors using the maximum likelihood method to check their structure. We sorted the factor consistency coefficients for each item by size, and after organizing the final factor types after optimal oblique rotation, we identified three factors for health attitudes and four for health behaviors. Secondly, we aimed to explore the relationships between knowledge, attitudes, and behaviors in health education at universities through path analysis using covariance structure analysis with the maximum likelihood method and Standardized estimates Covariance-based SEM(CB-SEM)model and Multi-Group SEM model was constructed. We repeatedly modified the model by first removing cross-load items and then deleting the four low-efficiency project paths until we achieved the best fit. We will determine the validity of the model using fit indices GFI (Goodness-of-Fit Index), AGFI (Adjusted Goodness-of-Fit Index), CFI (Comparative Fit Index) and RMSEA (Root Mean Square Error of Approximation). We judge that if GFI is greater than or equal to AGFI, the closer GFI and AGFI are to 1, the better the model fits; The CFI evaluates how much better your hypothesized model fits the data compared to a baseline “null model.” It ranges from 0 to 1, with higher values indicating better fit. RMSEA is an index that shows the deviation between the model distribution and the true distribution per degree of freedom, with a value less than 0.05 showing a very good fit, and a value of 0.1 or higher showing a poor fit (20). Finally, we reprocessed the data by combining the affirmative responses of options 1 and 2 for health knowledge, health attitudes, and health behaviors, and combined the negative responses from options 3 and 4. We then calculated the proportions and used chi-square analysis to look at the statistical differences based on gender and field of study, with *p* < 0.05 being considered statistically significant. Statistical analysis was conducted using IBM SPSS Statistics 26 and IBM SPSS Amos 26 data analysis software.

## 3 Results

### 3.1 Current status of KAB in health education in universities

The current situation of KAB in university health education is analyzed in depth from three aspects: overall, gender differences, and major differences, leading to the following conclusions.

#### 3.1.1 Current status of health knowledge (Table 2)

**Table 2:** Health knowledge (%).

Items	Total (%)	Gender (%)		*x* ^2^	*p*	Major of study (%)		*x* ^2^	*p*
		Male	Female			Physical education	Non-physical education		
Health lifestyle									
K_1_	81.5	84.6	78.6	6.785	0.009	93.6	79.1	21.854	<0.001
K_2_	90	89.9	90	0.001	0.976	94.7	89	5.543	0.019
K_3_	86.1	88.3	84	4.411	0.036	92.6	84.8	7.912	0.005
K_4_	84.2	82.6	85.7	1.978	0.16	83.5	84.3	0.082	0.775
Disease prevention									
K_5_	83.3	88.9	78.1	13.102	<0.001	95.4	70.9	13.269	<0.001
K_6_	57.5	65.1	50.3	25.019	<0.001	71.8	54.6	18.909	<0.001
K_7_	65	71.7	58.8	20.508	<0.001	73.4	63.4	6.943	0.008
K_8_	68	72	64.1	8.052	0.005	86.2	64.3	34.338	<0.001
Mental health									
K_9_	82.6	84.8	80.5	3.638	0.056	93.1	80.5	17.238	<0.001
K_10_	82.9	85	80.9	3.411	0.065	93.1	80.8	16.574	<0.001
K_11_	83.1	85.4	81	3.783	0.052	93.6	81	17.667	<0.001
K_12_	83.2	85	81.6	2.411	0.12	93.1	81.3	15.701	<0.001
Sexual and reproductive health									
K_13_	90.8	93.6	88.1	10.159	0.001	94.1	90.1	3.072	0.08
K_14_	98	98.2	97.8	0.24	0.624	97.3	98.1	0.432	0.511
K_15_	94.4	93.4	95.3	1.979	0.159	96.8	93.9	2.46	0.117
K_16_	89.7	89.6	89.8	0.019	0.891	89.9	89.7	0.008	0.927
Safety emergency and risk avoidance									
K_17_	71.7	72.6	70.9	0.408	0.523	81.4	69.8	10.436	0.001
K_18_	72	72.8	71.4	0.267	0.606	82.4	70	12.112	0.001
K_19_	72.2	72.9	71.6	0.272	0.602	81.4	70.4	9.43	0.002
K_20_	72.4	72.9	71.9	0.154	0.694	81.4	70.6	9.103	0.003

1. The overall awareness rate of health knowledge about “healthy lifestyles” is 81.5 to 90.0%. In the analysis of gender differences, significant differences were found in the items of methods to enhance physical fitness (K_1_) and the dangers of smoking (K_3_). Specifically, the awareness rate of health knowledge for males in the item methods to enhance physical fitness (K_1_) is 84.6%, higher than the 78.6% for females. In the item dangers of smoking (K_3_), the awareness rate for males is 88.3%, higher than the 84% for females. There are no statistical gender differences in the items of healthy dietary habits (K_2_) and knowledge of food safety (K_4_).
  In the analysis of major differences, significant differences were found in the items of methods to enhance physical fitness (K_1_), healthy dietary habits (K_2_), and the dangers of smoking (K_3_). The awareness rates of health knowledge among physical education students are significantly higher than those of non-physical education students. Specifically, the awareness rate for physical education students in methods to enhance physical fitness (K_1_) is 93.6%, significantly higher than the 79.1% for non-physical education students. In healthy dietary habits (K_2_), the awareness rate for physical education students is 94.7%, significantly higher than the 89.0% for non-physical education students. In the dangers of smoking (K_3_), the awareness rate for physical education students is 92.6%, significantly higher than the 84.8% for non-physical education students. No statistical differences were found in knowledge of food safety (K_4_) among different majors.
2. The overall awareness rate of health knowledge regarding “disease prevention” is 57.5%–83.3%. Among these, the awareness rate for flu prevention knowledge (K_5_) is the highest at 83.3%, while the awareness rate for the dangers of antibiotics to health (K_6_) is the lowest at 57.5%, which is also the lowest among all health knowledge items. In the analysis of disease prevention items, significant differences were found in flu prevention knowledge (K_5_), the dangers of antibiotics to health (K_6_), common methods to assess health status (K_7_), and knowledge of chronic disease prevention (K_8_) across gender and major groupings. In the gender grouping, all items in the health knowledge of “disease prevention” showed that males had a significantly higher awareness rate than females. In other words, males have a higher level of knowledge acquisition regarding disease prevention than females. This difference may be attributed to males being more interested in knowledge about certain diseases and having more opportunities to encounter such knowledge. In the major grouping, physical education students had a significantly higher awareness rate of health knowledge regarding “disease prevention” than non-physical education students. This shows that physical education programs in universities are better at promoting health education, while the training programs for non-physical education students do not emphasize health education.
3. The overall awareness rate of health knowledge about “mental health” is 82.6%–83.2%. No statistical gender differences were found in the awareness rate of health knowledge regarding “mental health.” However, in the major grouping, significant differences were found in the knowledge of depression and anxiety symptoms (K_9_), the relationship between mental health and physical health (K_10_), promoting positive emotions and alleviating negative emotions (K_11_), and ways to self-regulate anxiety and worries (K_12_), with physical education students showing a significantly higher awareness rate than non-physical education students. This also reflects that physical education students in universities have more comprehensive access to mental health knowledge than non-physical education students.
4. The overall awareness rate of health knowledge about “sexual and reproductive health” is 89.7%–98%. The overall awareness rate of health knowledge in this dimension is the highest compared to other dimensions. Among these, the awareness rate for knowledge of AIDS prevention (K_14_) reached 98%, the highest overall. This is due to the government’s long-term efforts in AIDS prevention and control. The knowledge awareness rate for methods to prevent sexual assault (K_13_) showed significant differences in the gender grouping, with males at 93.6% significantly higher than females at 88.1%. This phenomenon is quite special and requires attention. Generally, females are the primary targets of sexual assault and need to understand and master methods of prevention. However, 11.9% of females are not very familiar with methods to prevent sexual assault, which shows that education on preventing sexual assault in our country is still lacking and explains the frequent occurrence of sexual assault cases in recent years. No statistical differences were found in the awareness rates of all items in the major grouping.
5. The overall awareness rate of health knowledge regarding “safety emergency and risk avoidance” is 71.7%–72.4%. Compared to other aspects, this overall rate is relatively low. Although the state has emphasized this area sufficiently and most universities have taken corresponding measures regarding safety emergencies and risk avoidance, to achieve better results, we need to stay vigilant and not let our guard down (21). No significant differences were found in the awareness rates of health knowledge among all items in the gender grouping. However, in the major grouping, all items showed statistically significant differences. Physical education students had significantly higher awareness rates than non-physical education students in the four knowledge items of self-rescue and mutual rescue in drowning (K_17_), health and safety risks during travel (K_18_), emergency measures for animal bites and scratches (K_19_), and methods of cardiopulmonary resuscitation and trauma rescue (K_20_). Since sports activities also carry certain risks, physical education students engage in sports behaviors much more than non-physical education students, which significantly increases their probability of encountering safety risks, leading to a higher emphasis on safety in the daily education of physical education students.

#### 3.1.2 Current status of health education attitudes (Table 3)

**Table 3:** Rate of formation of attitudes toward health education (%).

Items	Total (%)	Gender (%)		*x* ^2^	*p*	Major of study (%)		*x* ^2^	*p*
		Male	Female			Physical education	Non-physical education		
Emotional willingness									
A_1_	95.0	95.1	95.0	0.002	0.961	96.3	94.8	0.741	0.389
A_2_	94.9	94.0	95.9	2.105	0.147	95.2	94.9	0.034	0.853
Learning willingness									
A_3_	98.5	97.6	99.3	5.392	0.020	97.9	98.6	0.582	0.455
A_4_	98.1	96.9	99.3	9.002	0.003	97.9	98.2	0.086	0.769
A_5_	98.8	98.5	99.1	0.890	0.345	98.4	98.9	0.387	0.534
A_6_	98.2	97.6	98.8	20,210	0.137	98.9	98.1	0.654	0.419
A_7_	96.6	96.2	97.1	0.712	0.399	95.2	96.9	1.388	0.239
A_8_	95.1	94.7	95.5	0.407	0.524	94.7	95.2	0.094	0.760
Value recognition									
A_9_	97.7	97.3	98.1	0.893	0.345	98.4	97.6	0.506	0.477
A_10_	96.5	96.2	96.7	0.261	0.610	94.1	96.9	3.492	0.062
A_11_	98	97.6	98.4	1.001	0.317	96.3	98.4	3.699	0.054
A_12_	99.5	99.5	99.5	0.005	0.943	99.5	99.5	0	0.999
A_13_	96.4	95.4	97.2	2.636	0.104	97.3	96.2	0.616	0.432
A_14_	98.8	98.4	99.3	2.255	0.133	99.5	98.7	0.765	0.382

Firstly, from the overall formation rate of health education attitudes, “emotional willingness” is 94.9%–95%, “learning willingness” is 95.1%–98.8%, and “value recognition” is 96.4%–99.5%. This indicates that college students have a very high emotional willingness and learning willingness towards learning health knowledge, as well as a good understanding of the value of health.

Secondly, from the perspective of gender grouping, significant differences were found in “learning willingness” regarding the importance of learning health knowledge for a healthy life (A_3_) and the importance of learning health knowledge (A_4_). Specifically, the formation rate of health education attitudes for the item regarding the importance of learning health knowledge for a healthy life (A_3_) is 99.3% for females, significantly better than 97.6% for males. The formation rate for the item regarding the importance of learning health knowledge (A_4_) is also 99.3% for females, significantly better than 96.9% for males. Even though females might not know as much about health as males, they have a better attitude toward health education. This also indicates that females have a stronger expectation for health education.

Finally, in the professional grouping, no statistical differences were found in the three dimensions of “emotional willingness,” “learning willingness,” and “value recognition,” indicating that college students’ attitudes toward health education are not influenced by their field of study.

#### 3.1.3 Current status of health behaviors (Table 4)

**Table 4:** How often health behavior is developed (%).

Items	Total (%)	Gender (%)		*x* ^2^	*p*	Major of study (%)		*x* ^2^	*p*
		Male	Female			Physical education	Non-physical education		
Proactive attention									
B_1_	93.8	93.8	93.8	<0.001	0.995	94.7	93.6	0.308	0.579
B_2_	97.9	97.6	98.1	0311	0.577	96.8	98.1	1.221	0.269
Proactive learning									
B_3_	92.2	90.3	94	5.233	0.022	91.5	92.3	0.155	0.694
B_4_	85.3	83.7	86.7	2.010	0.156	88.3	84.7	1.646	0.199
B_5_	93.1	92.3	93.8	0.946	0.331	97.3	92.2	6.361	0.012
B_6_	69.6	74.8	64.7	13.607	<0.001	84.6	66.6	24.009	<0.001
B_7_	91.9	92.7	91.2	0.831	0.362	96.8	90.9	7.248	0.007
Habit formation									
B_8_	89.4	93.6	85.3	20.171	<0.001	97.9	87.6	17.216	<0.001
B_9_	93	96.2	90	16.392	<0.001	98.9	91.8	12.238	<0.001
B_10_	93.9	94.5	93.3	0.735	0.386	95.7	93.5	1.369	0.242

The overall formation rate for “proactive attention” is 93.8% for actively paying attention to health knowledge (B_1_) and 97.9% for regularly checking food expiration dates (B_2_), showing a high formation rate. No statistical differences were found in gender and major of study grouping, suggesting that college students usually pay attention to health knowledge and food safety in their daily behaviors.

The overall formation rate for “proactive learning” is 69.6%–93.1%. You can see that the formation rates for some aspects of proactive learning in health education are relatively low, such as consulting teachers about health issues (B_6_) at only 69.6%, while other aspects have higher formation rates, such as self-learning health-related knowledge (B_5_) at 93.1%, showing a big difference. This is related to the current social context where college students are in an era of online information, leading to a fear of face-to-face communication. They find it more convenient and comfortable to search for answers online rather than consulting teachers. In the gender grouping, the formation rate for valuing flu prevention (B_3_) is 94% for females, significantly better than 90.3% for males. Research indicates that some males may underestimate the severity of flu and think it is not a big deal, thus not taking flu prevention seriously (22). The formation rate for consulting teachers about health issues (B_6_) is 74.8% for males, significantly better than 64.7% for females. According to studies, asking questions to teachers is a type of social activity, and research shows that boys are generally more proactive than girls during social activities. Mahesh et al. (23). In the professional grouping, significant differences were found in three items: self-learning health-related knowledge (B_5_), consulting teachers about health issues (B_6_), and browsing health information (B_7_), with health behavior formation rates for physical education students significantly higher than those for non-physical education students. This can be correlated with the higher health knowledge awareness rate among physical education students, suggesting that their comprehensive knowledge promotes the formation of health behaviors.

The overall formation rate for “habit formation” is 89.4%–93.9%. Significant differences were found in the gender and major of study grouping for the items of having a habit of exercising (B_8_) and consciously exercising to enhance physical fitness (B_9_), with males showing significantly higher health behavior formation rates than females. This indicates that males have an advantage in forming good health habits. In the professional grouping, health behavior formation rates for physical education students are significantly higher than those for non-physical education students. Physical education students generally have more regular exercise and health habits, and they recognize the importance of health. This result also suggests an internal correlation between the formation of health behaviors and health recognition attitudes.

### 3.2 KAB model of health education in colleges

To verify the internal correlation among knowledge, attitudes, and behaviors in college health education, data from health knowledge, health attitudes, and health behaviors were validated through structural equation modeling. The current status of health education KAB suggests that health knowledge has an internal correlation with health attitudes and health behaviors. After repeated attempts and optimization operations such as deleting paths, a SEM validation model was formed. The overall KAB model of health education in universities was ultimately obtained. Although the chi-square to degrees of freedom ratio is relatively large (*χ*^2^/df = 19.853), this may stem from the large sample size (*N* = 4,508). However, other fit indices performed excellently (GFI = 0.972, AGFI = 0.947, CFI = 0.982, RMSEA = 0.065), indicating that the overall fit of the model to the data is actually acceptable. The model validation results support the core hypothesis of KAB theory (Table 5).

**Table 5:** Evaluation of the measurement model.

Model fit	GFI	AGFI	CFI	RMSEA	χ^2^/df
Criteria	> 0.90 (acceptable) > 0.95 (excellent)	> 0.90 (acceptable) > 0.95 (excellent)	>0.90 (acceptable) > 0.95 (excellent)	< 0.08 (acceptable) < 0.05 (excellent)	–
Model Results	0.972	0.947	0.982	0.065	19.853
Evaluation	Excellent	Acceptable	Excellent	Acceptable	–

**Figure 1:**
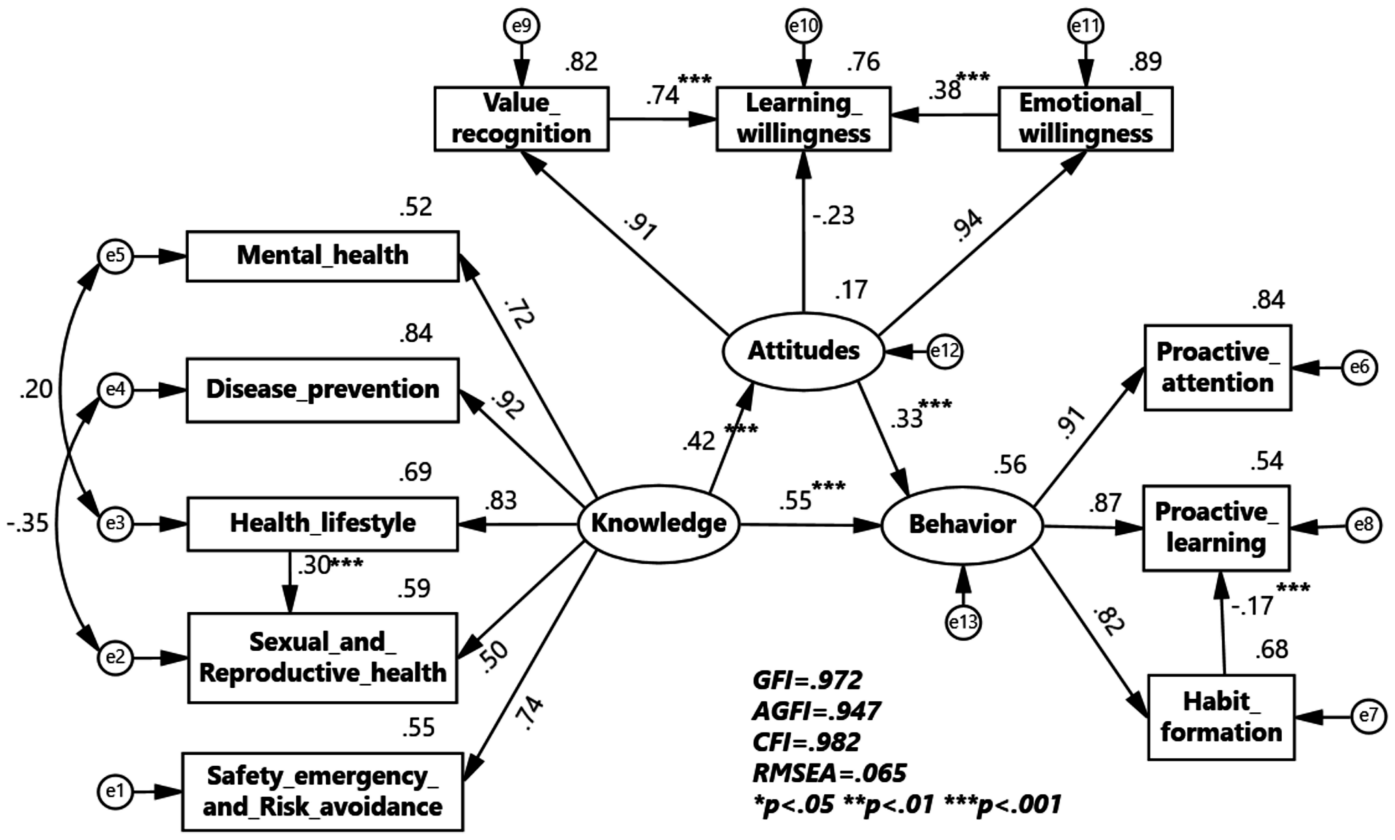
KAB model of Health Education in Colleges and Universities.

Additionally, the output results of the specific SEM are presented in Table 6. the path coefficients indicate that the relationship from college students’ health knowledge to health attitudes shows a positive causal relationship coefficient of 0.42, the relationship from health attitudes to health behaviors has a positive causal relationship coefficient of 0.33, and the relationship from health knowledge to health behaviors has a positive causal relationship coefficient of 0.55, all reaching statistically significant levels. This shows that health knowledge not only directly affects health behaviors but also helps improve them by boosting health attitudes. Furthermore, the link between a healthy lifestyle and sexual health has a positive effect of 0.30, indicating that acquiring knowledge about healthy lifestyles can influence knowledge about sexual and reproductive health. In health attitudes, the relationship between health value and learning willingness shows a strong positive causal relationship of 0.74, and the relationship between emotional willingness and learning willingness shows a positive causal relationship of 0.38. This indicates that the values and emotional perspectives regarding health education influence the willingness to learn health education. Notably, in health behaviors, the relationship between habit formation and proactive learning shows a weak negative causal relationship of 0.17, suggesting that once health education behaviors become habitual, they may affect proactive learning behaviors (Figure 1).

**Table 6:** Standardized regression weights.

Path		Standardized estimate	Unstandardized estimate	S.E.	C.R.	*p*
Knowledge	→ Health lifestyle	0.829	0.883	0.016	55.313	***
Knowledge	→ Disease prevention	0.919	1.197	0.020	59.995	***
Knowledge	→ Mental health	0.719	0.924	0.019	48.069	***
Knowledge	→ Sexual and reproductive health	0.496	0.460	0.028	16.180	***
Knowledge	→ Safety emergency and risk avoidance	0.739	1.000			
Health lifestyle	→ Sexual and reproductive health	0.303	0.264	0.024	11.150	***
Knowledge	→ Attitudes	0.415	0.379	0.015	25.156	***
Knowledge	→ Behavior	0.551	0.621	0.018	35.017	***
Attitudes	→ Emotional willingness	0.942	1.144	0.019	60.346	***
Attitudes	→ Learning willingness	−0.233	−0.226	0.157	−1.439	0.150
Attitudes	→ Value recognition	0.905	1.000			
Attitudes	→ Behavior	0.326	0.402	0.017	24.063	***
Value recognition	→ Learning willingness	0.742	0.654	0.055	11.884	***
Emotional willingness	→ Learning willingness	0.384	0.307	0.082	3.735	***
Behavior	→ Habit formation	0.823	0.829	0.013	63.178	***
Behavior	→ Proactive attention	0.914	1.000			
Behavior	→ Proactive learning	0.869	0.987	0.038	26.168	***
Proactive learning	→ Proactive learning	−0.169	−0.191	0.033	−5.821	***

***p < 0.001 (highly significant).

**Figure 2:**
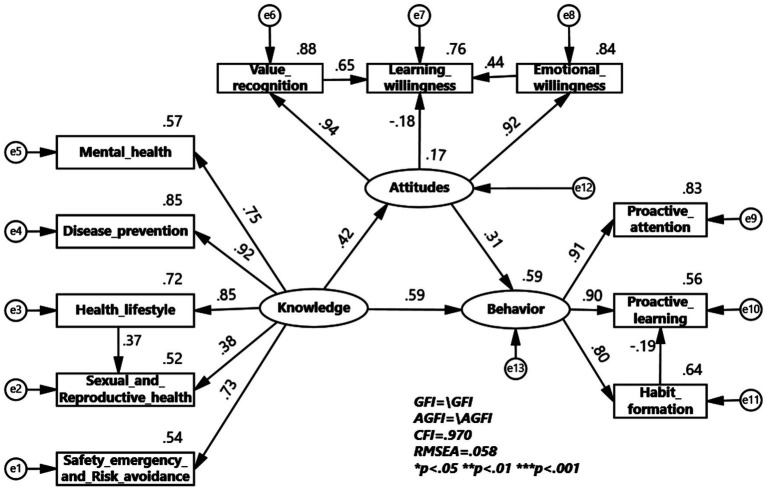
KAB model for Health Education Program among male.

**Figure 3:**
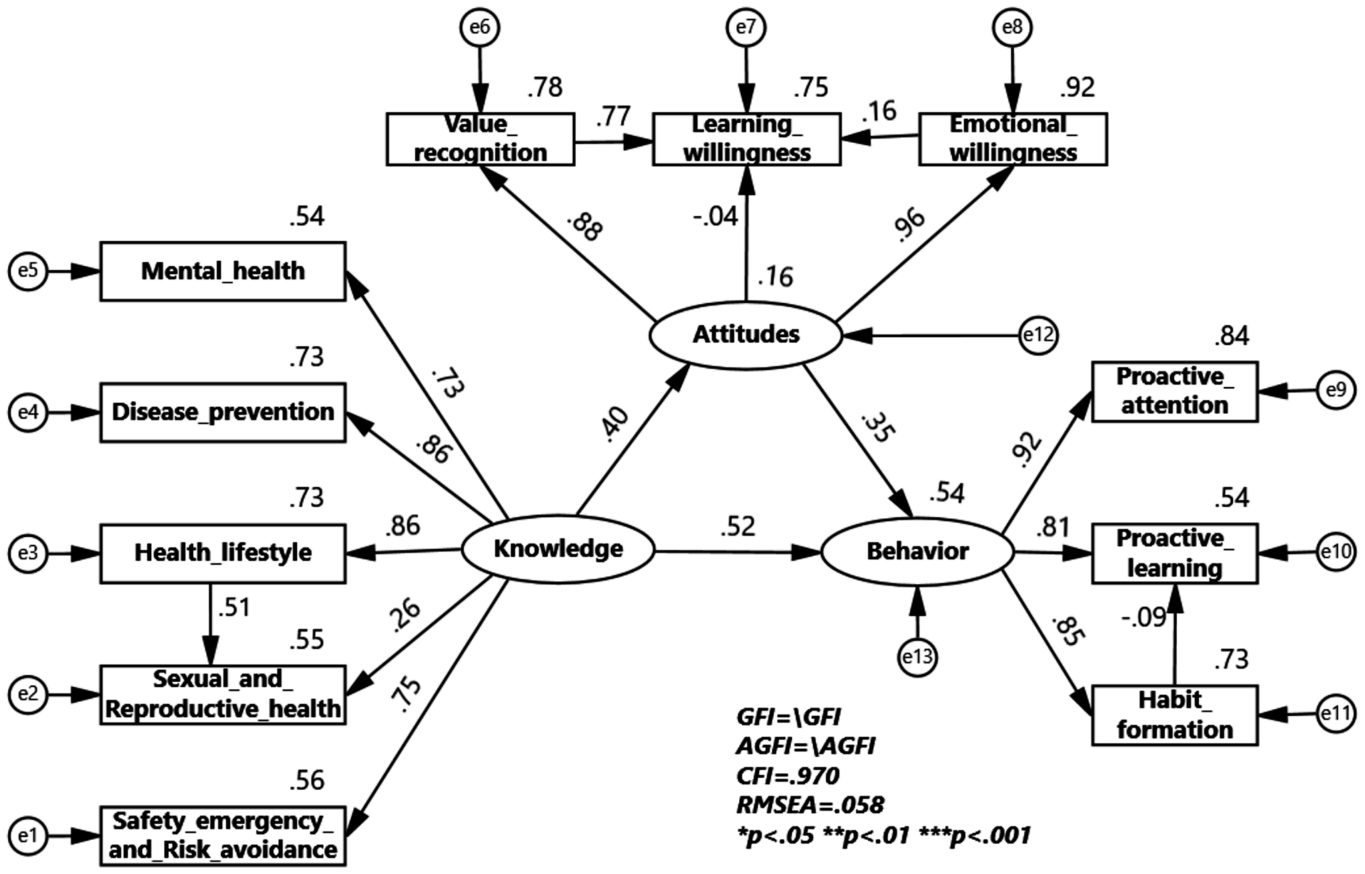
KAB model for Health Education Program among female.

To verify the universality of the KAB model of health education in colleges across different samples, multi-group structural equation model validation analyses were conducted for gender and major of study grouping. The multi-group structural equation model for the gender group shows that health knowledge has a greater impact on health attitudes and health behaviors in the male group, while health attitudes have a greater impact on health behaviors in the female group. The impact coefficient of healthy lifestyle on sexual and reproductive health is 0.37 for males, greater than 0.51 for females, indicating a larger impact in the male group. The impact coefficient of value recognition on learning willingness is 0.65 for males and 0.77 for females, indicating a greater impact in the female group. The impact coefficient of emotional willingness on learning willingness is 0.44 for males and 0.16 for females, indicating a larger impact in the male group. The impact coefficient of habit formation on proactive learning is −0.19 for males and −0.09 for females, indicating a larger impact in the male group (Figures 2, 3).

Through comparison with the hypothesized model, the multi-group validation analysis for the gender group shows that in the validation of six models constrained by Measurement weights, Measurement intercepts, Structural weights, Structural covariances, Structural residuals, and Measurement residuals, the values of NFI (Normed Fit Index), IFI (Incremental Fit Index), RFI (Relative Fit Index), and TLI (Tucker–Lewis Index) are all less than 0.05, proving that the constructed model has confirmatory validity across different gender variable samples (Table 7).

**Table 7:** Gender group validation analysis of gender categories.

Model	DF	CMIN	*p*	NFI delta-1	IFI delta-2	RFI rho-1	TLI rho2
Measurement weights	8	110.470	0.000	0.003	0.003	−0.001	−0.001
Measurement intercepts	19	460.862	0.000	0.013	0.013	0.005	0.005
Structural weights	26	594.588	0.000	0.016	0.016	0.005	0.005
Structural covariances	27	669.341	0.000	0.018	0.018	0.007	0.007
Structural residuals	29	641.317	0.000	0.017	0.017	0.005	0.005
Measurement residuals	40	846.498	0.000	0.023	0.023	0.005	0.005

**Figure 4:**
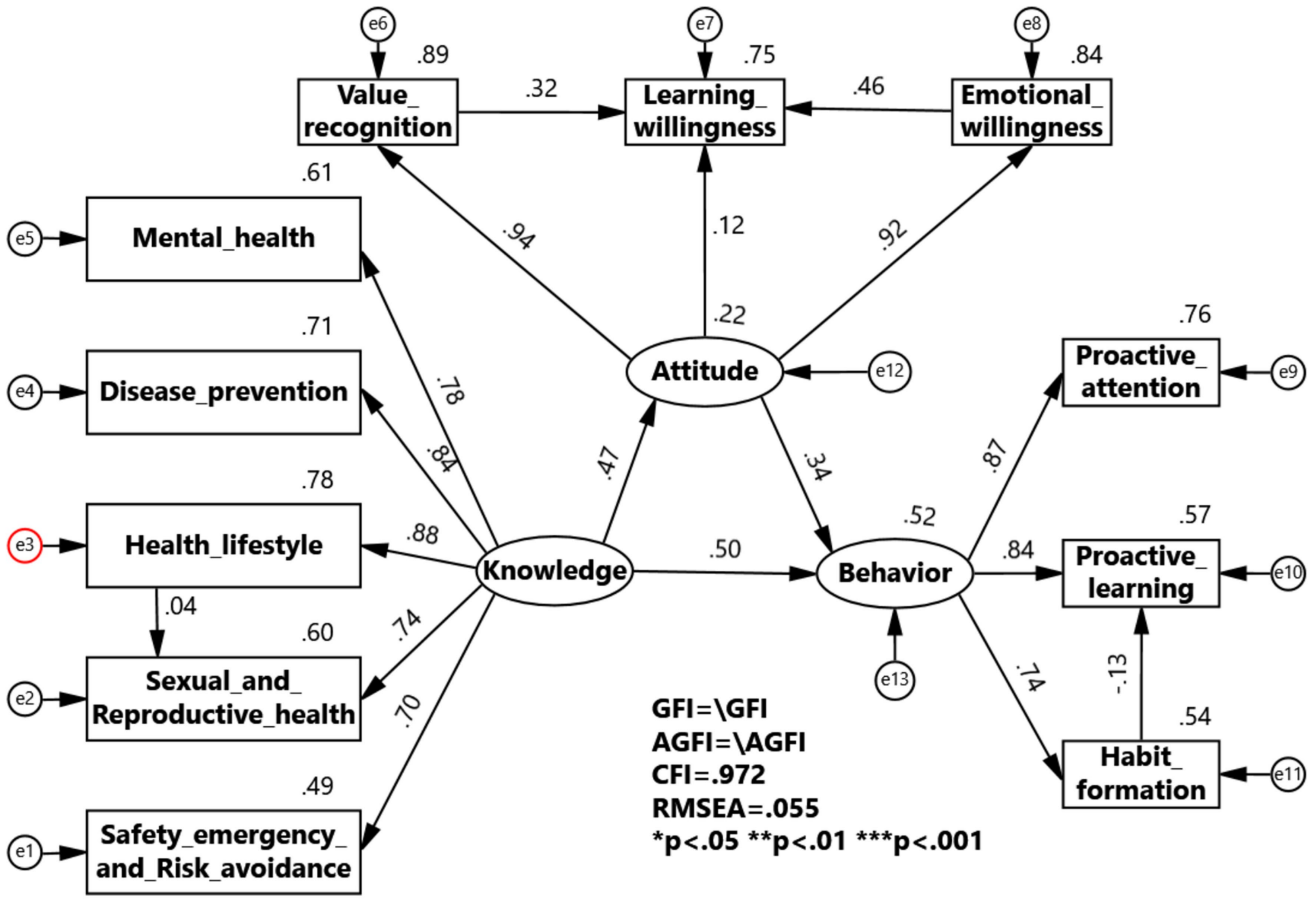
KAB model for Health Education Program among physical education.

**Figure 5:**
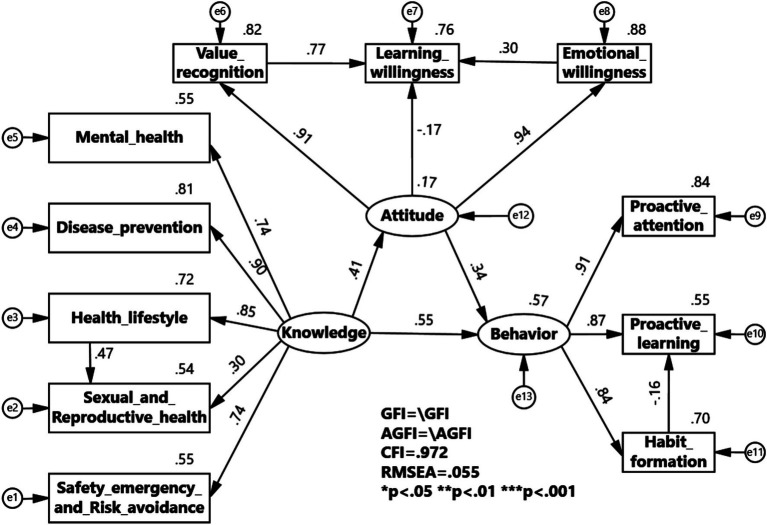
KAB model for Health Education Program among non-physical education.

The multi-group structural equation model for the professional grouping shows that health knowledge has a greater impact on health attitudes and health behaviors in the physical education group, while in the non-physical education group, health knowledge has a greater impact on health behaviors, with health attitudes having the same level of impact on health behaviors. The impact coefficient of healthy lifestyle on sexual and reproductive health is 0.04 for physical education students, which is less than 0.47 for non-physical education students, indicating a larger impact in the non-physical education group. The impact coefficient of value recognition on learning willingness is 0.32 for physical education students and 0.77 for non-physical education students, indicating a greater impact in the non-physical education group. The impact coefficient of emotional willingness on learning willingness is 0.46 for physical education students and 0.30 for non-physical education students, indicating a larger impact in the physical education group. The impact coefficient of habit formation on proactive learning is −0.13 for physical education students and −0.16 for non-physical education students, indicating a larger impact in the non-physical education group (Figures 4, 5).

Through comparison with the hypothesized model, the multi-group validation analysis for the professional grouping shows that in the validation of six models constrained by Measurement weights, Measurement intercepts, Structural weights, Structural covariances, Structural residuals, and Measurement residuals, the values of NFI, IFI, RFI, and TLI are all less than 0.05, proving that the constructed model has confirmatory validity across different professional variable samples (Table 8).

**Table 8:** Gender group validation analysis of gender categories.

Model	DF	CMIN	*p*	NFI delta-1	IFI delta-2	RFI rho-1	TLI rho2
Measurement weights	8	94.061	0.000	0.003	0.003	−0.001	−0.001
Measurement intercepts	19	428.308	0.000	0.012	0.012	0.005	0.005
Structural weights	26	600.872	0.000	0.016	0.016	0.006	0.006
Structural covariances	27	601.601	0.000	0.016	0.016	0.006	0.006
Structural residuals	29	624.873	0.000	0.017	0.017	0.006	0.006
Measurement residuals	40	681.994	0.000	0.019	0.019	0.002	0.002

## 4 Discussion

This study systematically reveals the complex associations and group heterogeneity of the knowledge-attitude-behavior (KAB) model in college health education. The results indicate that students in physical education majors exhibit comprehensive KAB advantages, with a significantly higher awareness rate of health knowledge (e.g., 15.7% higher in disease prevention compared to non-physical education majors) and a significantly higher formation rate of health behaviors (e.g., proactive learning reaching 93.1%), confirming the synergistic effect of integrating health education with professional courses. This finding aligns with Traoré et al.’s observations in schistosomiasis prevention and control, where targeted health training significantly improved the KAB levels of the target population (from 74.9% to 88.1% compliance) (24). However, the KAB shortcomings of non-physical education students (e.g., only 57.5% awareness of antibiotic dangers) highlight the structural deficiencies in current general health education, echoing Zhu et al.’s findings on low-education groups in chronic disease KAB studies (25).

In the gender dimension, this study observed a significant “cognitive-attitude inversion”: males excel in knowledge of healthy lifestyles (e.g., K_1_ awareness rate of 84.6% vs. female 78.6%) and knowledge of disease prevention and sexual assault defense (K_13_ male 93.6% vs. female 88.1%), while females are more proactive in health learning attitudes (e.g., A_4_ recognition of the importance of health knowledge at 99.3% vs. male 96.9%). This suggests that male college students generally have a better understanding of physical fitness and smoking than their female counterparts, which might be because males usually show more interest in sports and fitness in their daily lives (26). Regarding the dangers of smoking, research indicates that males perceive smoking as “masculine,” and the smoking rate among male college students is significantly higher than that of females (27–29). The apparent paradox of higher knowledge coinciding with higher smoking prevalence among males is resolvable within the integrated KAB-SEM framework. The significant K → B path indicates that knowledge is a potent antecedent, likely by elevating perceived threat (11). However, its effect on the ultimate behavior is likely suppressed by countervailing forces. For males, strong subjective norms that link smoking to masculine identity and positive social prototypes may override reasoned intentions (30, 31). Furthermore, a gender-inclined optimistic bias, potentially exacerbated by high knowledge, may lead to a discounting of personal susceptibility (12). This suggests that for young males, smoking initiation and maintenance may be less a failure of knowledge and more a function of social reactivity and perceived invulnerability. Future interventions should therefore target these gendered normative perceptions and cognitive biases rather than focusing solely on knowledge dissemination. This contradiction partly stems from differences in risk exposure, with higher smoking rates and exercise participation among males, but it is crucial to note the relative lack of defense knowledge among high-risk female groups (32). Particularly concerning is the phenomenon where males surpass females in knowledge of sexual assault prevention, challenging the conclusion from oral health KAB studies that “females generally pay more attention to health,” exposing gaps in current sexual education that do not accurately cover high-risk populations. This shortcoming needs to draw on China’s experience in HIV prevention and control, through sustained policy investment and health education (e.g., this study found an HIV awareness rate of 98%) to specifically strengthen these areas (24). Since the first report of AIDS cases in China in 1985, the central government has required all regions and departments to carefully study and formulate prevention and control plans, clarify relevant policies, conduct health education, implement prevention and control measures, and strengthen patient treatment (33).

The structural equation model further reveals a dual-pathway for KAB transformation: health knowledge not only directly drives behavior (*β* = 0.55) but also indirectly influences it through attitudes (knowledge → attitude *β* = 0.42, attitude → behavior *β* = 0.33), confirming the chain effect of health education. However, caution is warranted regarding the negative pathway of “habit formation inhibiting proactive learning” (*β* = −0.17), indicating that behavior solidification may weaken the willingness to explore. This phenomenon resonates with findings in digital health tool research, where individuals with high eHealth literacy rely more on preset information and reduce proactive learning (34). Notably, the knowledge integration effectiveness of physical education students (e.g., the path coefficient of healthy lifestyle promoting sexual health cognition at 0.30) validates the value of modular education, but this group is weaker in attitude-driven behavior (value recognition → learning willingness *β* = 0.32) compared to non-physical education students (*β* = 0.77), suggesting that mere knowledge transmission is insufficient for sustaining long-term behavior change.

The limitations of this study require careful consideration: (1) using just one regional sample makes it hard to apply these findings to other groups; (2) people might have answered the questionnaires in a way they thought looked good, such as the reported rate of safety emergency knowledge (71.7%) potentially being higher than actual (35); (3) confounding factors such as family health literacy were not controlled. Future research should look at how the KAB scale was developed for newborn hearing screening in South Africa to validate the robustness of the KAB model across multi-center samples (36); simultaneously, combining health behavior tracking technologies (e.g., wearable devices) to keep track of how habits and learning affect each other will provide empirical support for overcoming behavioral inertia. Practically, a “three-dimensional intervention system” should be constructed: targeting knowledge gaps through specialized courses in high-risk areas (e.g., antibiotic resistance simulation experiments), utilizing AI health partners to optimize accessibility of consultations for the internet generation, and designing gamified challenge tasks to reverse negative pathways of habits. Only through deep integration of cognitive reinforcement, technological empowerment, and behavioral activation can the gap between knowledge and action in college health education be bridged.

## Data Availability

The original contributions presented in the study are included in the article/supplementary material, further inquiries can be directed to the corresponding author.
